# FKBP52 dictates the dual role of p53 in hepatocellular carcinoma: Mutation-specific prognostic stratification and therapeutic implications

**DOI:** 10.1016/j.jbc.2026.113390

**Published:** 2026-08-05

**Authors:** Jinfeng Wu, Chengyan Tang, Zewu Meng, Weihong Chen, Li Zhu, Xiang Li, Yaoyu Liu, Xing Wang

**Affiliations:** 1 Key Laboratory of Gastrointestinal Cancer (Ministry of Education), School of Basic Medical Sciences, Fujian Medical University, Fuzhou, Fujian, P.R. China; 2 The Department of Hepatic Surgery, Fujian Medical University Union Hospital, Fuzhou, P.R. China; 3 Fujian Key Laboratory of Tumor Microbiology, Department of Medical Microbiology, Fujian Medical University, Fuzhou, P.R. China; 4 NHC Key Laboratory of Etiological Epidemiology of Chronic Diseases with High Incidence in Fujian-Taiwan Area (Co-construction), Fujian Medical University, Fuzhou, P.R. China

**Keywords:** hepatocellular carcinoma, p53, p53 mutation, FK506-binding protein, oncogenesis

## Abstract

The lack of mutation-contextual therapeutic targets remains a major barrier in hepatocellular carcinoma (HCC) management. Immunohistochemical analysis of 341 human HCC specimens identified FK506-binding protein 52 (FKBP52) as a tumor grade-associated biomarker. *In vitro*, FKBP52 overexpression enhanced proliferation in p53-mutant liver cancer cells but suppressed it in p53-wild-type cells. Using two murine models—subcutaneous xenografts and orthotopic liver implants—genetic manipulation confirmed that FKBP52 overexpression in Huh7 accelerated tumor growth, while shRNA-mediated knockdown attenuated growth and decreased AFP/CD133 expression. Differential p53 status underpinned this disparity. *In vitro* EdU incorporation assays, colony formation assays and TP53-null xenografts (Hep3B cells) demonstrated that p53 knockdown abrogated the phenotype, confirming that FKBP52 promotes mutant p53-driven tumorigenesis while suppressing wild-type p53 tumors. Mechanistically, co-immunoprecipitation (Co-IP) revealed FKBP52 binds p53 *via* its FK2 and TPR domains independently of mutation status. This interaction recruited MDM2 into the FKBP52-p53 complex, impairing MDM2-mediated degradation and prolonging stability of both wild-type and mutant p53. Taken together, our findings establish FKBP52 as a novel p53 stabilizer with context-dependent oncogenic effects, critically determined by p53 mutational status. These results highlight the therapeutic potential of targeting the FKBP52-MDM2-p53 axis specifically in TP53-mutant HCC.

Hepatocellular carcinoma (HCC) accounts for approximately 80% of all primary liver tumors and ranks as the sixth most common and fourth deadliest malignancy globally (1, 2, 3). Despite advances in treatment, overall survival among patients with HCC remains low (4, 5). HCC is characterized by its insidious onset and high recurrence rate after resection, underscoring the urgent need for improved early prognosis and risk stratification (3). While the Barcelona Clinic Liver Cancer (BCLC) staging system provides the best prognostic information for HCC patients (3), available biomarkers exhibit low sensitivity and heterogeneous specificity, even with varying cut-off points (6, 7, 8, 9). For instance, in a follow-up survey of HCC patients undergoing hepatectomy, the sensitivity of serum alpha-fetoprotein (AFP) levels was less than 34.1%, highlighting its limited prognostic value (6). Other biomarkers, such as circulating tumor DNA (ctDNA) (7) and cells (8), or extracellular vesicles (9), have been explored for HCC surveillance. However, their outcomes have been inconsistent, necessitating the continued search for more reliable biomarkers.

The FK506-binding protein (FKBP) family has emerged recently as attractive and versatile candidates in the treatment of various cancers (10, 11, 12). FKBPs belong to the immunophilin family, which bind the immunosuppressive drugs FK506 and rapamycin, and possess peptidyl-prolyl cis/trans-isomerase (PPIase) activity (13, 14). FKBP52 encoded by *FKBP4* gene, overexpress in hormone-dependent cancers and interestingly, abundant in the dysplastic hepatocytes of woodchuck hepatitis virus-driven transgenic mice (15, 16, 17, 18).

In this study, we examined FKBP52 expressions in different morphological subtypes obtained from liver cancer patients, comparing them pairwise with non-tumor liver tissues, and with focal nodular hyperplasia and liver cirrhosis. Using a large cohort, we demonstrate that FKBP52 is a promising prognostic biomarker, with its expression correlating with HCC grades and exhibiting specificity for HCC. This association has significant pathological implications. FKBP52 influences the growth characteristics of HCCs in a context-dependent manner by targeting either wild-type p53 or its mutant counterpart, which dominates the cell at the time. The *TP53* gene, often referred to as the “guardian of the genome,” is the most frequently mutated tumor suppressor in human cancers (19, 20). In some cancers, the mutant allele of p53 coexists with the wild-type allele until the loss of heterozygosity occurs, leading to the dominance of the mutant allele (21, 22). We identified that FKBP52 forms ternary complexes with p53 and its negative regulator, MDM2 (murine double minute 2) (19). By decreasing MDM2 expression, FKBP52 prevents p53 degradation. Consequently, the absence of FKBP52 in hepatic benign nodules may result in insufficient production of protective wild-type p53. In contrast, increased FKBP52 expression during HCC progression potentiates the accumulation of “hyperstable” mutant p53, which is critical for gain-of-function (GOF) effects. The ratio of mutant to wild-type p53 determines the manifestation of dominant-negative effects in sensitive cells (23, 24, 25). Thus, the functional outcome of FKBP52 depends on the mutational status of p53 during HCC stages, highlighting FKBP52 as an upstream regulator of p53 and a promising therapeutic candidate.

## Results

### FKBP52 is increased in human HCC and is associated with HCC grade

**Figure 1:**
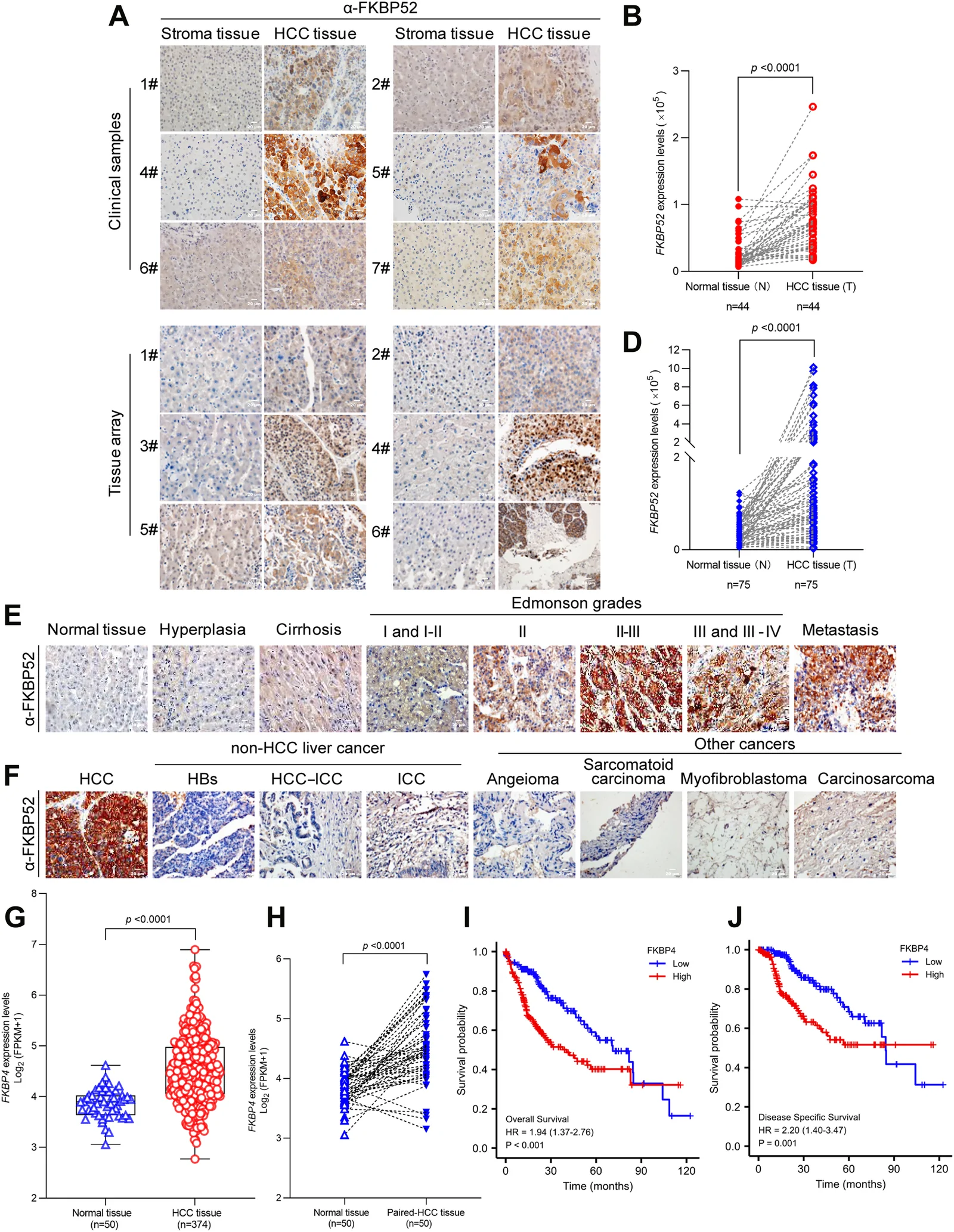
**FKBP52 is a promising biomarker for HCC prognostic diagnosis.***A*, FKBP52 staining in human primary HCC and peri-tumoral samples. Forty-four paired samples were analyzed; six representative pairs are shown. *B*, quantification of FKBP52 staining intensity in panel (*A*). *C*, representative picture of 75 tissue array-based paired HCC samples showing FKBP52 staining. *D*, quantification of staining intensity in panel (*C*). *E*, FKBP52 staining during HCC progression. Human liver biopsies in tissue microarrays were analyzed. See also Fig. S1. *F*, FKBP52 staining in cancer tissues other than HCC, including hepatoblastoma (HBs), mixed liver cancer (HCC-ICC) and intrahepatic cholangiocarcinoma (ICC). *G*, quantification of *FKBP4* mRNA expression in HCC tissues (n = 374) and unpaired nontumor tissues (n = 50) in the TCGA data set. *H*, quantification in paired HCC tissues (n = 50) in the above data set. *I*, Kaplan–Meier plot for overall survival in HCC patients from the data set in (*G*). *J*, Kaplan–Meier plot of disease-specific survival from the data set in (*G*). Scale bars denote 20 μm. Data are presented as mean ± SD. Two-tailed Student’s *t* tests were used in (*B*, *D*, *G*, and *H*). The log-rank Mantel-Cox test was performed in (*I* and *J*).

To quantify the levels of FKBP52 in human liver cancer, 341 biopsy tissue samples, including normal liver, hyperplastic livers, liver cirrhosis, HCCs, and other diseases such as intra-hepatic cholangiocarcinoma (ICC), were stained with anti-FKBP52 antibody (Fig. 1, *A*–*F*). Comparison of 44 paired normal and HCC samples confirmed elevated levels of FKBP52 in HCC. Under normal conditions, human hepatocytes, which comprise 70% of the liver parenchyma, expressed FKBP52 at low levels in the nuclei. However, FKBP52 was significantly upregulated in HCC tissues, where it was predominantly localized in the cytoplasm of hepatocytes (Fig. 1, *A* and *C*). Quantitative analysis revealed that the integrated optical density (IOD) of FKBP52 was 71,293 in HCC tissues compared to 18,058 in paratumorous tissues (*p* < 0.0001) (Fig. 1*B*). Notably, even within the same tissue, FKBP52 levels were higher in transformed hepatocytes than in less malignant cells and declined in syncytial giant cells (Fig. S1*A*).

Three tissue microarrays comprising 224 human liver biopsies, including normal, benign, malignant (with different grades), and metastatic liver tumors, were analyzed. FKBP52 levels were significantly higher in HCC tissues compared to paired normal tissues (*p* < 0.0001) (Fig. 1, *C* and *D*). Importantly, FKBP52 staining intensity was low in normal liver and hyperplasia tissues but elevated in Edmondson grades II and II-III of HCC, declining in metastatic tissues (Fig. 1*E*). The most significant *p*-value was observed in grade II (Fig. S1*B*). To assess specificity, 16 biopsy tissues from other liver diseases and 13 other types of cancer were stained. FKBP52 was either undetectable or present at very low levels in non-HCC cancer tissues compared to HCC tissues (Fig. 1*F*).

Analysis of the International Cancer Genome Consortium and The Cancer Genome Atlas (TCGA) revealed significantly higher levels of *FKBP4* mRNA in 374 human HCC samples with diverse etiologies and geographical origins (Fig, 1, *G* and *H*). Patients with higher *FKBP4* gene expression exhibited shorter overall survival and disease-specific survival (Fig. 1, *I* and *J*). Collectively, these findings suggest that FKBP52 is a potential prognostic biomarker specifically correlated with HCC and HCC grade.

### Conflicting roles of FKBP52 in hepatocarcinogenesis

**Figure 2:**
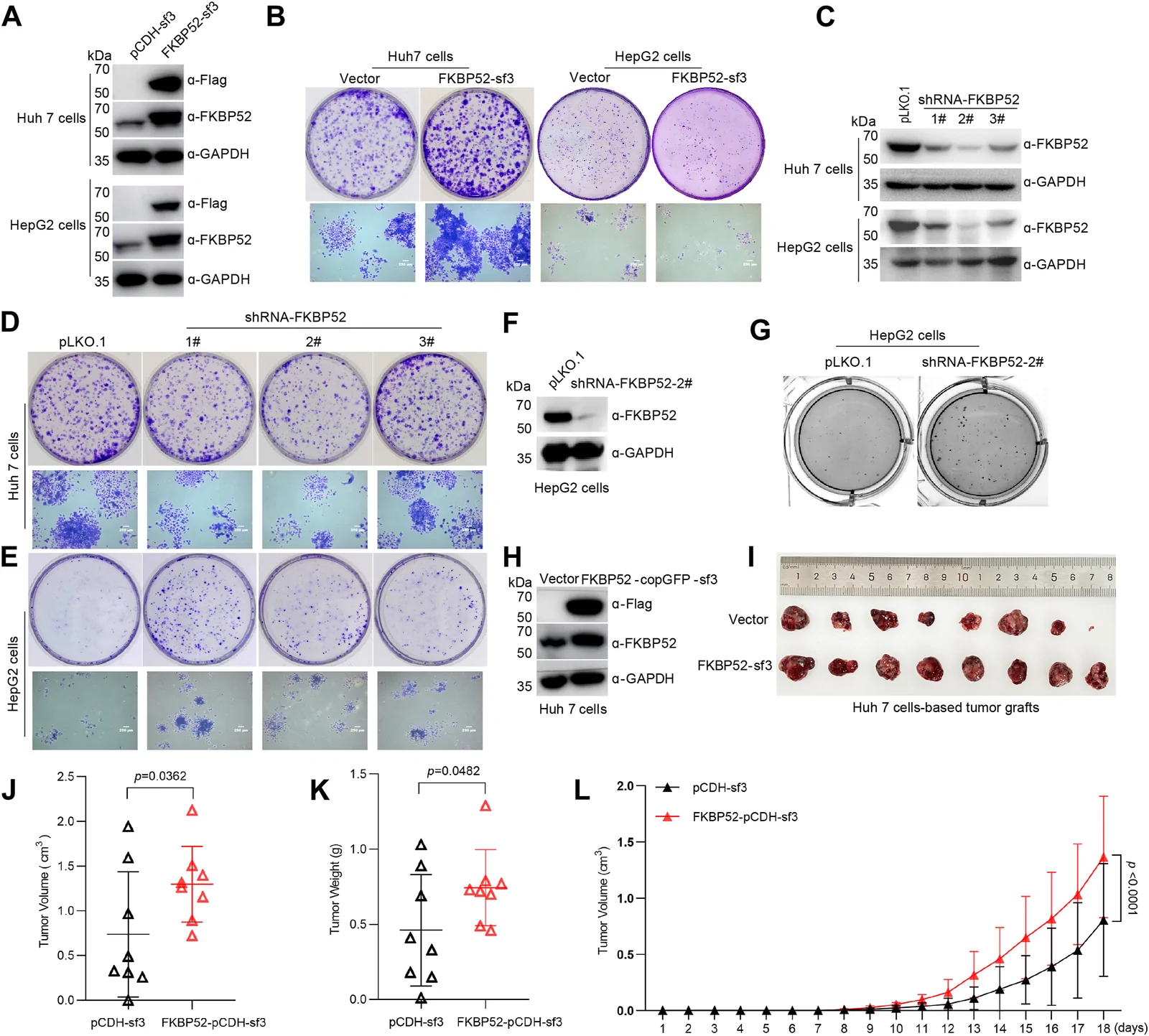
**The contradictory role of FKBP52 in cell type-dependent hepatocarcinogenesis.***A*, immunoblot of FKBP52-pCDH-sf3 expression in Huh 7 or HepG2 cells. *B*, representative images of colonies. *C*, immunoblot of knock down of FKBP52 protein in cells. *D* and *E*, representative images in colony formation assay for Huh 7 (*D*) and HepG2 cells (*E*). *F*, immunoblot of knock down of FKBP52 protein in HepG2 cells for soft agar assay. *G*, representative images for anchorage-independent growth of HepG2 cells in (*F*). *H*, immunoblot analysis of Huh 7 cells which express FKBP52-pCDH-copGFP-sf3 plasmid. *I*, excised subcutaneous tumor xenografts (n = 8) from the cells in (*H*). *J* and *K*, tumor volume (*J*) and weight (*K*) of the grafts in (*I*). They were measured at the end of the experiment. Tumor volume was calculated by the formula: (length × width^2^)/2. *L*, tumor growth curve indicated by tumor volume (cm^3^) which measured at indicated time points. The scale bar is 250 μm. Data were shown as mean ± s.e.m.; Two-tailed Student’s t tests were used in (*J* and *K*). Two-way ANOVA were used in (*L*).

To investigate the role of FKBP52 in HCC tumor growth, we performed a series of *in vitro* and *in vivo* experiments using two widely used liver cancer–related cell models, Huh7 and HepG2, derived from hepatocellular carcinoma and hepatoblastoma, respectively. FKBP52 was stably overexpressed using the FKBP52-pCDH-sf3 plasmid (Fig. 2*A*). Protein levels were quantified and presented in Figure S2*A*. Ectopic expression of FKBP52 significantly enhanced Huh7 cell proliferation but dramatically inhibited growth in HepG2 cells (Fig. 2*B*). Quantitative analysis showed that FKBP52 overexpression doubled the cell numbers compared to vector control and increased the absolute colony area to over 100,000 pixels/μm^2^ in Huh7 cells (Fig. S2, *B* and *C*). Conversely, RNA interference (RNAi)-mediated knockdown of FKBP52 reduced proliferation in Huh7 cells while markedly enhancing it in HepG2 cells, with shRNA-FKBP52 2# exhibiting the strongest knockdown efficiency (Figs. 2, *C*–*E* and S2*D*), as confirmed by quantitative analysis (Fig. S2, *E* and *F*). As HepG2 cells are poorly tumorigenic in immunosuppressed mice (26), we employed the soft-agar colony formation assay to assess their proliferative potential. As shown in Figures 2, *F* and *G* and S2, efficient knockdown of FKBP52 markedly increased the clonogenic capability of HepG2 cells. Notably, neither FKBP52 overexpression nor its inhibition affects the cell viability of Huh7 or HepG2 cells (Fig. S3, *A–D*).

**Figure 3:**
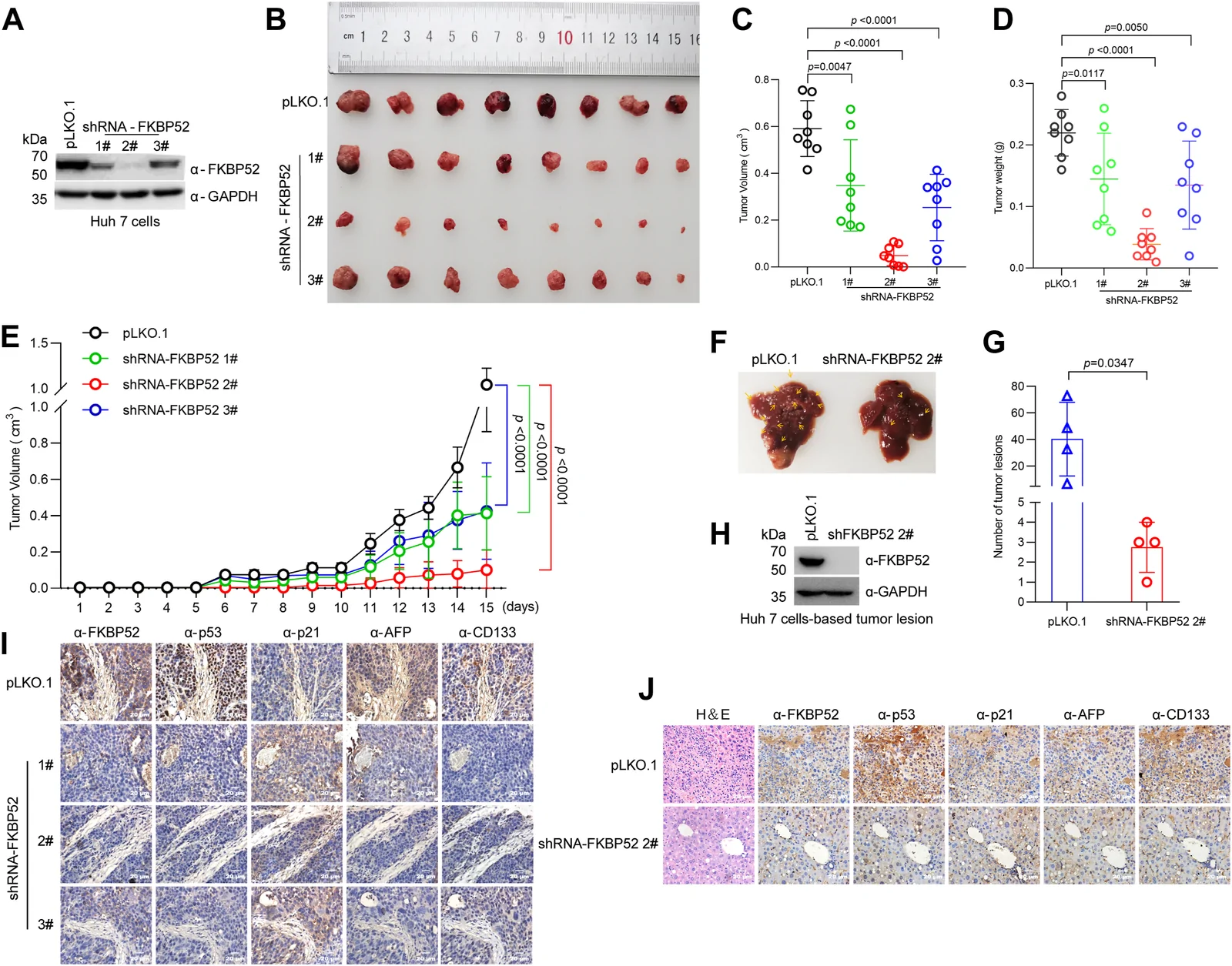
**FKBP52 is required for Huh 7 cell-based tumor growth *in vivo*.***A*, immunoblot analysis of Huh 7 cells stably expressing shRNA-FKBP52s. *B*, subcutaneous xenografts (n = 8) induced by cells in (*A*). *C* and *D*, tumor volume (*C*) and weight (*D*) in (*B*). Tumor volume was calculated as in Figure 2*J*. *E*, tumor growth curve indicated by tumor volume (cm^3^) which measured at indicated time points. *F*, gross images of tumor burden in the livers of mice after 8 weeks, which induced by orthotopic liver injections. Yellow arrowheads point to tumor nodules (n = 4). *G*, comparison of the number of tumor lesions on the liver surface. *H*, immunoblot analysis of FKBP52 expression in liver tumor lesions in (*F*). *I*, immunohistochemistry images of subcutaneous xenografts from (*B*). *J*, histology and immunohistochemistry images for liver tissues from (*F*). Tissues were probed for indicated antibodies. Representative images are shown. Scale bars denote 20 μm. Data were shown as mean ± s.e.m.; One- or Two-way ANOVA were used in (*C*), (*D* and *E*). Two-tailed Student’s t-tests were used in (*G*).

To confirm the oncogenic potential of FKBP52 *in vivo*, we established two mouse models (1): subcutaneous xenografts and (2) orthotopic liver injections. Lentiviral overexpression of full-length FKBP52 in Huh7 cells significantly accelerated tumor formation compared to vector controls (Fig. 2, *H–L*). FKBP52 overexpression induced malignant lesions as early as 11 days post-injection, with significant increases in tumor size (1.8-fold, *p* < 0.05) and weight (1.6-fold, *p* < 0.05) (Fig. 2, *J*–*L*). Conversely, shRNA-mediated FKBP52 knockdown (shRNA 2# showing maximal suppression, Fig. 3*A*) dramatically inhibited tumor growth, reducing average tumor size and weight to ∼60% and 35% of controls (*p* < 0.0001) (Fig. 3, *B*–*E*). In liver orthotopic xenograft models, Huh7 cells were injected at a depth of 3 to 8 mm in mouse liver for 8 to 10 weeks. Mice with lentiviruses carrying vector control displayed increased abdominal girth and livers with tumors. In contrast, FKBP52 knockdown mice had very limited tumor burden (*p* < 0.05; Fig, 3, *F* and *G*). Enumeration of the number of tumor lesions on the liver surface revealed an average number of 2.75 tumors in the shRNA-FKBP52 2# group, *versus* 40.5 upon vector treatment (Figs. 3*G* and S4*A*). The suppression effect of shRNA-FKBP52 2# was confirmed on tumor xenografts by > 90% (Fig. 3*H*).

The divergent effects of FKBP52 in Huh7 and HepG2 cells may be attributed to their p53 mutation status. HepG2 cells express wild-type p53, while Huh7 cells harbor a mutant p53 (Y220C, resulting from a tyrosine-to-cysteine substitution at amino acid 220) that is transcriptionally inactive yet aberrantly stable (27). Histological analysis of orthotopic tumors revealed that FKBP52 knockdown significantly reduced the number of tumor foci, as confirmed by low-magnification (10×) views of entire liver sections (Fig. S4*B*). This was accompanied by decreased expression of mutant p53 protein, as well as the HCC biomarkers AFP and CD133 (28, 29) (Figs. 3, *I* and *J* and S4*B*). Interestingly, the increased expression of the gene encoding p21 (*CDKN1A*) was identified (Fig. 3*I*), which is the canonical transcriptional target for p53 that arrests the cell cycle (30, 31). Our results were consistent with research in which inhibition of the aggregation of cancer-associated mutant p53 will restore the tumor suppressor function of p53 (32). The upregulation of p21 was not evident in the orthotopic model, which may be due to the discrepancy in shRNA-FKBP52 efficacy between the models (Fig. 3*J*). These findings collectively demonstrate that FKBP52 stabilizes mutant p53 in HCC, and its inhibition depletes oncogenic mutant p53 pools, thereby suppressing tumor growth. This highlights FKBP52 as a novel regulator of the p53 pathway in HCC.

### FKBP52 drives tumorigenesis in cell-derived xenograft models in a p53-dependent manner

**Figure 4:**
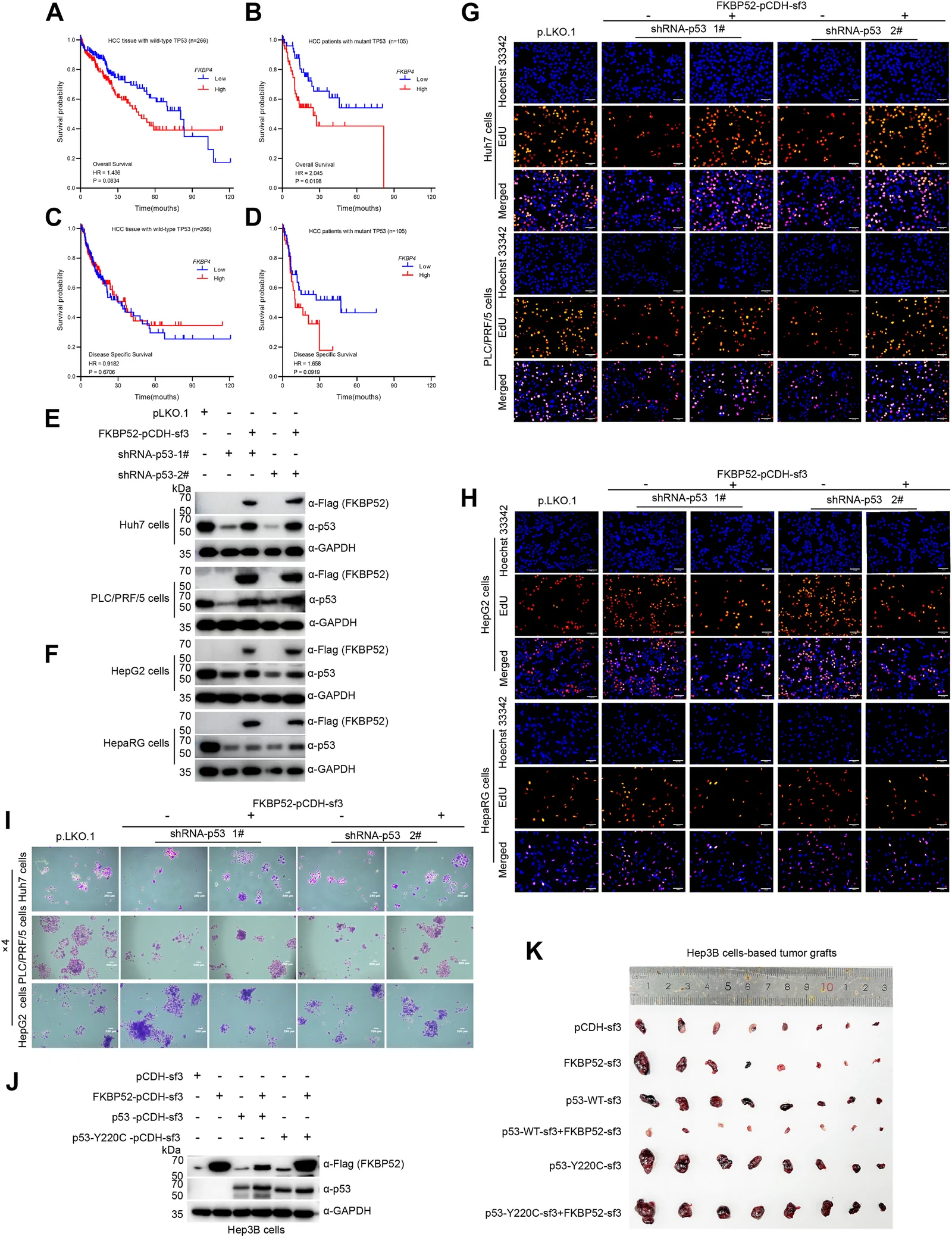
**p53 mutation status is dependent on FKBP52’s effect in oncogenesis.***A* and *B*, Kaplan–Meier plot for overall survival by *FKBP4* expression in HCC patients expressing wild-type *TP53* (n = 266) (*A*) or mutant *TP53* (n = 105) (*B*). *C* and *D*, Kaplan–Meier plot of disease-specific survival in (*A* and *B*). *E* and *F*, immunoblot analysis of FKBP52 overexpression and p53 knockdown in Huh7 and PLC/PRF/5 cells (*E*), and HepG2 and HepaRG cells (*F*). *G* and *H*, representative images of EdU incorporation assays in cells described in (*E* and *F*). *I*, representative images of colonies by cells in (*E* and *F*). *J*, immunoblot of wild-type p53 or Y220C-p53 overexpression in Hep3B cells, or upon co-transfection with FKBP52. *K*, subcutaneous tumor xenografts (n = 8) formed by Hep3B cells in (*J*). The scale bar is 100 μm, 250 μm. Representative images are shown. The log-rank Mantel-Cox test was performed in (*A–D*). All experiments were repeated at least three times.

To investigate the possible role of p53 genotype in FKBP52-mediated oncogenesis, we analyzed TCGA data. Our analysis revealed that HCC patients harboring mutant *TP53* with high *FKBP4* expression exhibited significantly shorter overall survival compared to those with wild-type *TP53* (Fig. 4, *A* and *B*). This trend was particularly pronounced in disease-specific survival, where mutant *TP53* patients with elevated *FKBP4* expression showed dramatically shortened survival times (Fig. 4, *C* and *D*). Notably, high FKBP52 expression was also associated with shortened OS in p53-wild-type patients (Fig. 4*A*). However, as OS is confounded by non-cancer-related deaths and subsequent therapies, we additionally analyzed progression-free interval (PFI) and disease-free interval (DFI) as surrogate endpoints that more directly reflect tumor-related events, such as progression, recurrence, new tumor events, and tumor-associated death, and are thus more sensitive to tumor-intrinsic biology (33). These analyses revealed that FKBP52 is strongly associated with PFI and DFI specifically in patients with TP53-mutant HCC (Fig. S5, *B* and *D*), but not in those with wild-type TP53 (Fig. S5, *A* and *C*). Collectively, these findings indicate that the TP53-dependent oncogenic impact of FKBP52 is more faithfully captured by recurrence-related and disease-specific endpoints than by OS, further validating the pathological relevance of the FKBP52–p53 interaction in HCC progression.

To determine whether p53 is required for FKBP52-mediated oncogenic effects, we performed p53 knockdown alone or in combination with FKBP52 overexpression in p53-mutant (Huh7 and PLC/PRF/5) and p53-wild-type (HepG2 and HepaRG) cells. PLC-PRF-5 is a HBV-positive hepatocellular carcinoma cells with c-myc overexpression and TP53 mutation (R249S) (34). Given the lack of widely recognized HCC cell lines with wild-type p53, we included HepaRG, an immortalized hepatic cell line that retains many characteristics of primary human hepatocytes (35), as a complementary model. shRNA-mediated p53 knockdown effectively reduced endogenous p53 protein levels in both p53-mutant and wild-type cells (Fig. 4, *E* and *F*). Notably, FKBP52 overexpression partially restored p53 expression in all cells (Fig. 4, *E* and *F*). Quantification of band intensities is provided in Figure S6, *A* and *B*.

To further assess the functional relevance of this regulatory interplay, we performed EdU incorporation assays to examine DNA synthesis during S phase. In p53-mutant Huh7 (Y220C) and PLC/PRF/5 (R249S) cells, p53 knockdown reduced the proportion of EdU-positive cells, that was markedly reversed by FKBP52 overexpression, indicating a pro-proliferative role for FKBP52 in the mutant p53 context (Fig. 4*G*). Conversely, in p53-wild-type HepG2 and HepaRG cells, p53 knockdown significantly increased EdU incorporation, whereas FKBP52 overexpression attenuated it, resulting in a substantial reduction in DNA synthesis compared with p53-silenced cells (Fig. 4*H*). Quantification of EdU-positive cells is provided in Figure S6, *C* and *D*. Collectively, these findings support a TP53-status-dependent role of FKBP52 in HCC cell proliferation.

Consistent with these findings, p53 knockdown reduced colony formation in p53-mutant cells, an effect reversed by co-expression of FKBP52-pCDH-sf3. In contrast, in p53-wild-type HepG2 cells, the increased colony formation induced by p53 knockdown was suppressed by FKBP52 overexpression (Fig. 4*I*). Due to the highly differentiated phenotype and slower proliferation rate, HepaRG cells exhibited limited colony formation efficiency, in contrast to Huh7, HepG2, and PLC/PRF/5 cells with higher clonogenic potential (data not shown). Original magnification images and quantitative analyses are provided in Figure S6, *E*–*G*.

To *in vivo* validate the p53-dependent nature of FKBP52's oncogenic role, we employed Hep3B cells—a p53-null hepatoma model with homozygous *TP53* deletion in nude mice (36) (Figs. 4*J* and S6*H*). Interestingly, stable individual overexpression of either FKBP52 or p53 in Hep3B cells promoted tumor growth, which may be related to the activation of other signaling pathways. Strikingly, stable FKBP52 overexpression exerted dichotomous effects depending on p53 status: it suppressed tumor growth in wild-type p53-reconstituted Hep3B cells while promoting proliferation in cells expressing the oncogenic mutant p53-Y220C (Fig. 4*K*), a finding consistent with our conclusion.

The functionally opposing outcomes in Figure 4 conclusively demonstrate that FKBP52 acts as a dual-context regulator in HCC, exerting pro-tumorigenic effects in p53-mutated environments but tumor-suppressive activity in p53-wild-type settings. This p53 genotype-dependent duality not only solidifies FKBP52's role as a contextual modulator of p53 signaling but also reveals its capacity to function as either an oncogene or tumor suppressor based on TP53 mutational status. Importantly, our findings position FKBP52 upstream of p53 in HCC pathogenesis, suggesting its potential as a therapeutic target for tailoring precision strategies in TP53-mutant HCC.

### FKBP52 stabilizes p53 independent of its mutational status

**Figure 5:**
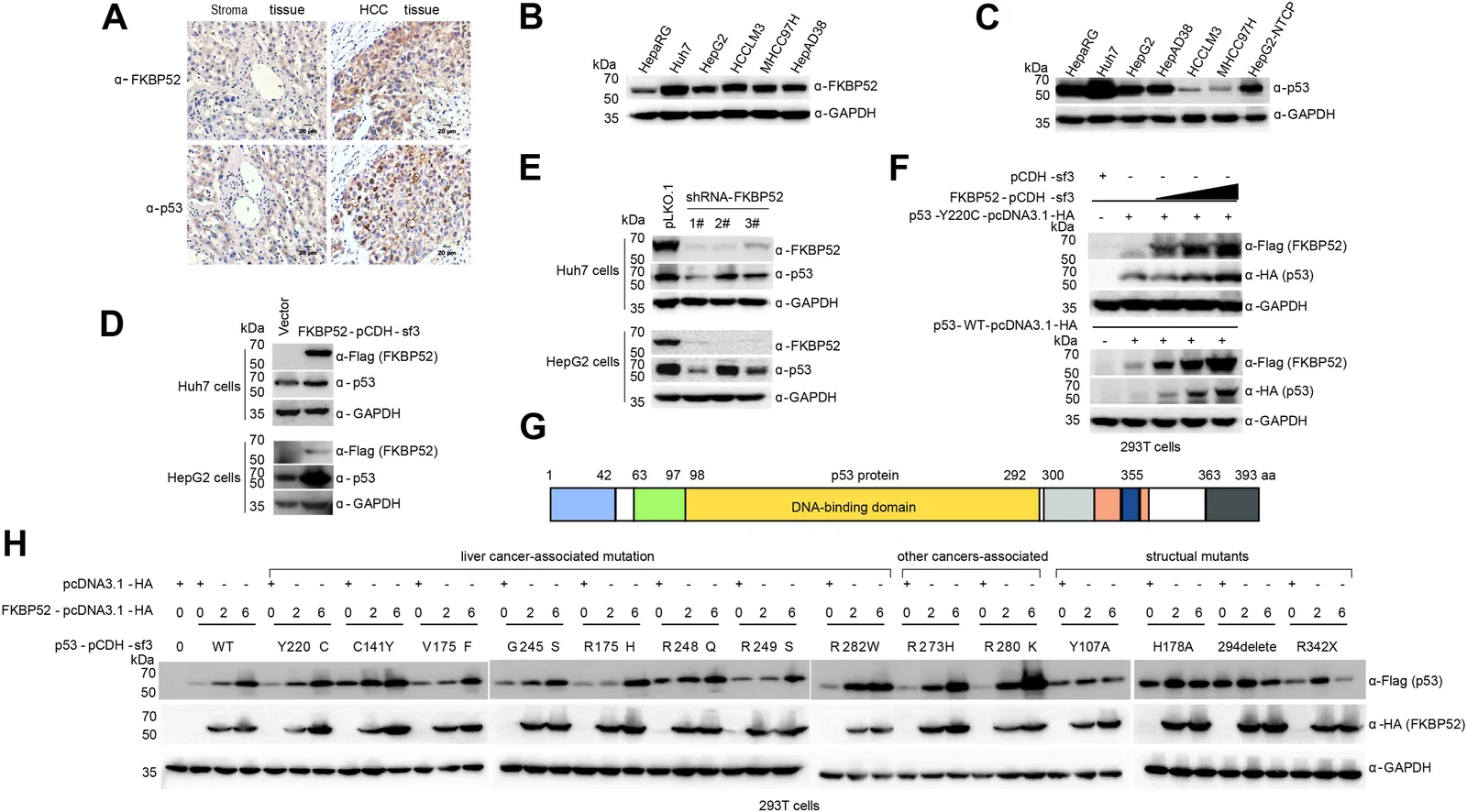
**FKBP52 is required for p53 protein level independent of the mutation.***A*, FKBP52 and p53 staining in paired HCC samples. *B* and *C*, immunoblot analysis of FKBP52 (*B*) and p53 (*C*) in various hepatocyte or HCC cell lines. *D*, immunoblot analysis of ectopic FKBP52 to endogenous p53 in Huh7 and HepG2 cells. *E*, immunoblot of knockdown of FKBP52 to p53 in the two cell lines. *F*, immunoblot analysis of FKBP52 dosage to p53 Y220C and wild-type plasmid in 293T cells. *G*, schematic illustration of p53 structure. *H*, immunoblot of FKBP52 dosage to wild-type p53 and the 14 mutants in 293T cells. Cell lysates were probed for the indicated antibodies. Scale bars denote 20 μm. Representative images are shown. All experiments were repeated at least three times.

Immunohistochemical analysis of human HCC tissues revealed elevated expression of both FKBP52 and p53 compared to adjacent normal liver (Fig. 5*A*). Consistent with this, analysis of the same batch of cell lysates revealed that human hepatic cell lines (Huh7) exhibited higher FKBP52 and p53 levels than non-transformed HepaRG cells (Fig. 5, *B* and *C*). Quantification of band intensities is provided in Figure S7, *A* and *B*. To directly assess FKBP52's regulatory role, genetic manipulation experiments revealed that FKBP52 overexpression increased endogenous p53 levels both in Huh7 and HepG2 cells. However, in contrast to the stable transfection of shRNA-FKBP52, where shRNA2# exhibited the maximal knockdown effect, shRNA1# demonstrated the strongest FKBP52 knockdown in transient transfection, which reduced p53 expression by approximately 70% in both cell lines (Fig. 5, *D* and *E*). Quantification of band intensities is provided in Figure S7, *C* and *D*.

To exclude cell line-specific effects, we ectopically expressed wild-type p53 (cloned from HepG2) and mutant Y220C (derived from Huh7) in non-liver-derived 293T cells. FKBP52 overexpression enhanced p53 protein levels in a dose-dependent manner, demonstrating mutational status-independent regulation (Fig. 5*F*). Strikingly, this stabilizing effect extended to 14 clinically prevalent p53 hotspot mutants (including Y220C, R249S, R175H; full list in Figure 5*G* (37, 38, 39, 40), where FKBP52 increased mutant p53 levels by 1.3- to 8.0-fold (*p* < 0.05 for all variants) and wild-type p53 by 3.1-fold (*p* < 0.01) across tested doses (Fig. 5*H*).

**Figure 6:**
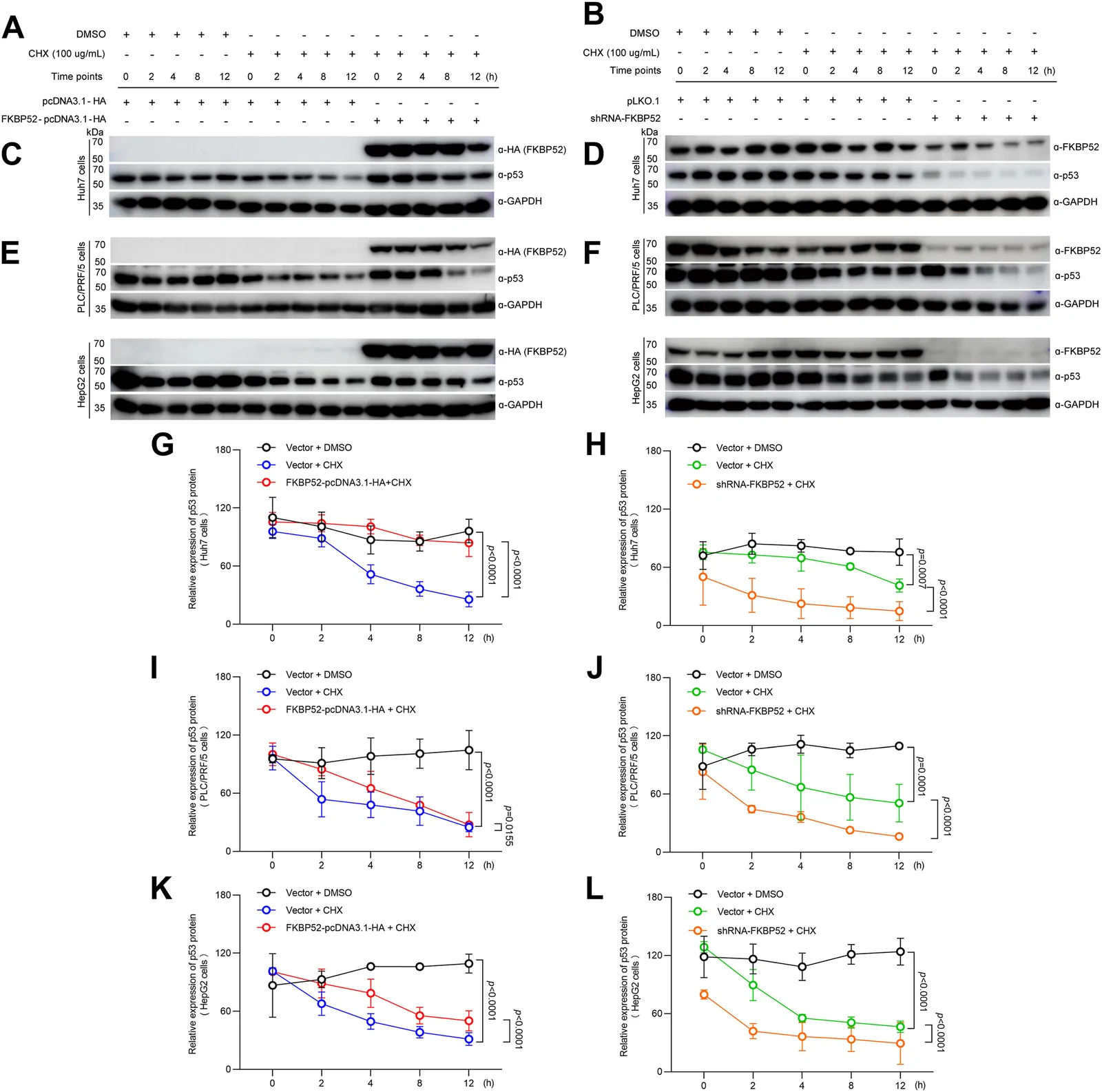
**FKBP52 prolongs the half-life of p53 protein.***A–F*, representative immunoblots of p53 stability following FKBP52 overexpression or shRNA-mediated knockdown in Huh7 (*A* and *B*), PLC/PRF/5 (*C* and *D*), and HepG2 (*E* and *F*) cells. *G–L*, densitometric quantification of p53 protein levels from panels *A–F*, normalized to GAPDH. Data are presented as mean ± SD from at least three independent experiments and analyzed by two-way ANOVA (*G–L*).

To determine whether FKBP52 regulates p53 stability, we performed cycloheximide (CHX) chase assays. In mutant-p53 cells of Huh7 and PLC/PRF/5, FKBP52 overexpression extended p53 half-life, while FKBP52 knockdown by using a shRNAs pool accelerated p53 degradation (Fig. 6, *A*–*D*, *G*–*J*). Similar results were observed in HepG2 cells: FKBP52 prolonged wild-type p53 half-life, whereas FKBP52 inhibition reduced it (Fig. 6, *E*, *F*, *K*, and *L*). These findings demonstrate that FKBP52 stabilizes p53 protein independently of its mutational status, revealing a novel regulatory mechanism in HCC.

### FKBP52 interacts with p53 and attenuates MDM2-mediated degradation

**Figure 7:**
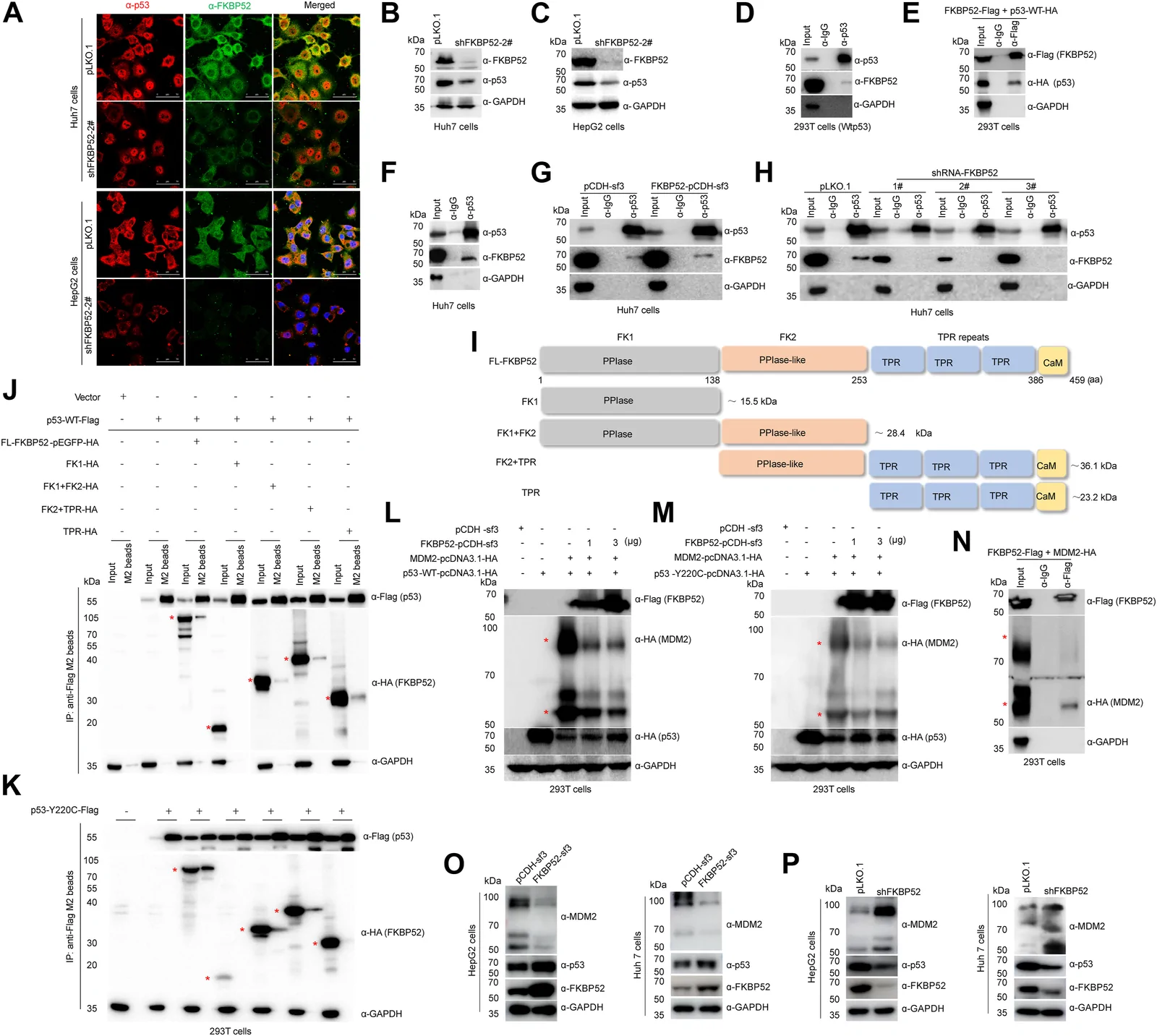
**FKBP52 interacts with and stabilizes p53 by preventing MDM2-mediated degradation.***A*, immunofluorescent images of FKBP52 and p53 co-localization. Cells were stained by DAPI. *B* and *C*, immunoblot analysis in (*A*). *D*, immunoblot analysis of immunoprecipitation by α-p53 in wild-type p53 harboring 293T cells. E, immunoblot analysis of immunoprecipitation by α-Flag (FKBP52) in 293T upon co-transfection. *F*, immunoblot analysis of α-p53-mediated immunoprecipitation in Huh 7 cells. *G*, immunoblot analysis of α-p53-mediated immunoprecipitation in Huh 7 cells, but upon FKBP52 overexpression. *H*, immunoblot of immunoprecipitation upon shRNA-FKBP52s. *I*, schematic illustration of full-length FKBP52 and the mutants. *J and K*, immunoblot of M2 magnetic beads-mediated immunoprecipitation in 293T upon co-transfection with WT (*J*) and Y220C-p53 (*K*) with full-length FKBP52 and the truncations. Red asterisks point to the plasmid expression. *L* and *M*, immunoblot of WT (*L*) and Y220C-p53 (*M*) p53 upon co-transfection with ectopic MDM2 and FKBP52. *N*, immunoblot of α-Flag (FKBP52)-mediated immunoprecipitation upon the co-transfection with MDM2-overexpressing plasmid. *O* and *P*, immunoblot analysis of MDM2 following FKBP52 overexpression or knockdown in cells. Cells were probed for indicated antibodies and representative images were shown. Scale bars denote 50 μm. All experiments were repeated at least three times.

To elucidate the mechanism underlying FKBP52-mediated p53 stabilization, we first investigated their physical interaction using complementary approaches. Confocal microscopy revealed cytoplasmic co-localization of FKBP52 with both wild-type and mutant p53, which was significantly diminished upon FKBP52 inhibition (Fig. 7, *A*–*C*). Consistent with this spatial association, co-immunoprecipitation (Co-IP) assays confirmed endogenous binding between FKBP52 and wild-type p53 in 293T cells (Fig. 7*D*), with reciprocal interactions observed under ectopic expression (Fig. 7*E*). Similar binding was identified in Huh7 cells with mutant p53 (Y220C) (Fig. 7, *F* and *G*), and FKBP52 knockdown reduced these interactions (Fig. 7*H*), confirming that FKBP52 binds p53 independently of its mutational status.

To pinpoint FKBP52's p53-binding domains, we performed structural domain analysis (ref. (16), Fig. 7*I*) followed by systematic pull-down assays. Notably, full-length FKBP52 and its FK2+TPR (aa 139–459) or TPR (aa 254–459) fragments—but not the FK1 domain (aa 1–138)—bound both wild-type and mutant p53 (Fig. 7, *J* and *K*), identifying the FK2 and TPR domains as critical interaction modules.

Given FKBP52's lack of intrinsic E3 ligase activity, we investigated its regulatory effect on MDM2, the primary ubiquitin ligase targeting p53 for degradation (41, 42). FKBP52 overexpression dose-dependently reduced levels of both MDM2-FL (∼90 kDa; 70% reduction at the highest dose) and MDM2-B (∼50 kDa; 52% reduction) isoforms (Figs. 7, *L* and *M* and S8, *A* and *B*). Intriguingly, Co-IP assays revealed FKBP52 specifically associates with the 57 kDa MDM2-B isoform (Fig. 7*N*), which lacks p53-binding capacity but dimerizes with MDM2-FL to enhance its ligase activity (43). The results were verified in liver cancer cell lines. FKBP52 overexpression consistently reduced MDM2 and increased p53 across all cell lines (Figs. 7*O* and S8*C*), whereas FKBP52 knockdown led to the opposite effects (Figs. 7*P* and S8*D*). Based on these findings, we propose a model wherein FKBP52 recruits MDM2-B to form an FKBP52-p53-MDM2-B ternary complex, thereby sequestering MDM2-FL and inhibiting its proteasomal degradation of p53—a novel regulatory mechanism explaining FKBP52's mutation-agnostic stabilization of p53.

## Discussion

The identification of stage-specific biomarkers is critical for guiding clinical management in aggressive malignancies like HCC. While FKBP52 has been implicated in hormone-dependent pathologies, its role in hepatocarcinogenesis remains poorly understood (44). Our study, leveraging 341 clinical samples, establishes FKBP52 as a diagnostic and prognostic biomarker for HCC, with expression at a very low level in Edmondson grade I tumors but peaking in advanced stages. This biphasic expression pattern suggests FKBP52 exerts stage-specific functional roles during HCC progression.

Central to this duality is FKBP52’s context-dependent interaction with p53. We demonstrate that FKBP52 acts as an oncogene in p53-mutant HCC by stabilizing mutant p53, while paradoxically suppressing tumorigenesis in p53-wild-type contexts. Genetic models revealed that FKBP52 knockdown enhanced proliferation in wild-type p53 cells of HepG2 and HepaRG, but inhibited growth in mutant-p53 cells of Huh7 (Y220C) and PLC/PRF/5 (R249S). Critically, FKBP52 rescued p53 loss-of-function phenotypes in both models, positioning p53 as the molecular switch governing FKBP52’s functional outcomes. These findings suggest a model in which low FKBP52 levels in premalignant lesions permit wild-type p53 loss, while its upregulation in advanced tumors drives mutant p53 accumulation—enabling dominant-negative or gain-of-function effects that fuel progression.

Mutant p53 acquires oncogenic properties through hyperstability (45), which we show is directly facilitated by FKBP52 *via* two mechanisms (1): physical interaction through its FK2 and TPR domains (independent of its peptidyl-prolyl isomerase activity) and (2) suppression of MDM2-mediated degradation. This stabilization mirrors the amyloid-driven hyperstability observed in other cancers, where mutant p53 aggregates correlate with malignancy grade (46). Intriguingly, FKBP52 knockdown not only reduced HCC biomarkers (AFP/CD133) but also upregulated p21 (30, 31)—a tumor-suppressive p53 target—suggesting partial restoration of wild-type p53 function. These parallels position FKBP52 as a therapeutic target to counteract mutant p53-driven malignancy, akin to oligopyridylamide-based strategies that disrupt mutant p53 aggregates.

While FKBP52’s FK2 and TPR domains mediate p53 stabilization, its FK1 domain—which is implicated primarily in protein misfolding in other contexts (47)—are dispensable for this interaction. This functional decoupling contrasts with FKBP52’s role in estrogen receptor α (ERα) regulation, where prolyl isomerase activity governs stability (16). Whether FKBP52 directly catalyzes p53 mutations or stabilizes pre-existing mutants independent of MDM2 warrants further investigation.

In conclusion, our work repositions FKBP52 as a molecular rheostat in HCC, with dual roles dictated by p53 mutation status. Its stage-specific expression and functional plasticity highlight its potential as both a prognostic biomarker and a therapeutic target. For p53-wild-type HCC, FKBP52 inhibition may enhance tumorigenesis, whereas in p53-mutant cases, targeting FKBP52 could destabilize oncogenic mutant p53—a strategy with broad implications for precision oncology.

## Experimental procedures

### Human tissues

Forty-four pairwise paraffin-embedded tissues were collected. Each pair comprised HCC tissue and adjacent normal liver tissue. Non-pairwise samples comprised of eight HCC and eight non-tumor tissues. All HCC samples were collected from patients with a pathological diagnosis. This clinical study was conducted in accordance with the Declaration of Helsinki principles and approved by the Institutional Review Board of Fujian Medical University, Fuzhou, China. Three tissue microarrays (TMAs) were purchased from Shanghai Xinchao Biological Technology Co, Ltd: HLivH150CS05, HLivDis060PT01, and HLivH060CD03. They contained 237 tissue samples. The sample set were deidentified and were considered exempt from patient consent by the Institutional Review Board. Data from The Cancer Genome Atlas Liver Hepatocellular Carcinoma (TCGA-LIHC; https://portal.gdc.cancer.gov/projects/TCGA-LIHC) were analyzed. Patients were stratified into FKBP4-high and FKBP4-low groups based on FKBP4 expression levels. Overall survival (OS), disease-specific survival (DSS), disease-free interval (DFI), and progression-free interval (PFI) were evaluated using Kaplan–Meier analysis with log-rank tests, including stratification by TP53 status.

### Mouse models

All animal work was approved by Institutional Animal Care and Use Committee (IACUC) of Fujian Medical University. Nude mice purchased from Shanghai Laboratory Animal Co., Ltd were used for all *in vivo* experiments. Livers of 5- to 8-week-old male mice were analyzed for hepatocarcinogenesis parameters. To establish a cell line-derived xenograft model, 5 × 10^6^ Huh 7 cells or Hep3B cells in 0.2 ml serum-free Dulbecco’s modified Eagle’s medium (DMEM, Invitrogen) containing 50% matrigel (40183 ES, YESEN) were injected subcutaneously into the flanks of mice. Two to 3 weeks after injection, when tumors were > 1000 and < 2000 mm^3^ in size, or when body weight had declined by > 20%, mice were euthanized according to Rutgers IACUC guidelines. When the mice were sacrificed, all tumors were collected, weighted, and photographed. Tumor size was measured using calipers and tumor volume (V) was calculated as (W^2^ × L)/2, where W is tumor width and L is tumor length. To establish an orthotopic mouse model, 1 × 10^5^ Huh 7 cells resuspended in 20 μl DMEM containing 50% matrigel were surgically implanted into the left lobe of the liver through a midline-mediated abdominal incision in 8-week-old male mice. After 8 to 10 weeks, necropsy was performed, intrahepatic sites of tumor were noted, photographed, counted, and samples were harvested for immunohistochemistry.

### Cell lines

Huh 7 (RRID:CVCL_0336), HepG2 (RRID:CVCL_0027), Hep3B (RRID:CVCL_0326), HCCLM3 (RRID:CVCL_6832), and MHCC-97H (RRID:CVCL_4972), PLC/PRF/5 (RRID:CVCL_0485) and the HEK293T (RRID:CVCL_0063) cell lines were purchased from the Cell Bank of Type Culture Collection, Chinese Academy of Sciences. Immortalized HepaRG, HepG2-hNTCP, and HepAD38 cells were kind gifts from Professor Qiang Deng (Fudan University). The cells were cultured in DMEM supplemented with 10% fetal bovine serum (FBS), 100 U/ml penicillin, and 100 μg/ml streptomycin at 37 °C in a 5% CO_2_ humidified incubator, unless otherwise indicated. All cell lines were tested for *mycoplasma* contamination.

### Plasmids

For the construction of vectors, Flag-, hemagglutinin (HA)- or GFP-tagged full-length FKBP52 (1–459 amino acids [aa], M23263.1) and its truncated constructs FK1 (1–138 aa), FK1+FK2 (1–253 aa), FK2+TPR (138–459 aa), and TPR (253–459 aa) were established by cloning the corresponding fragments into the pCDH-CMV-copGFP-puro-sf3 (Systems Biosciences) or pcDNA3.1-HA vectors (Invitrogen). The full-length cDNA of FKBP52 and wild-type p53 was amplified by PCR from the cDNA from HepG2 cells using PrimeSTAR Max DNA Polymerase (R045A, TaKaRa Bio). The mutant p53 of tyrosine at position 220 aa to cysteine (Y220C), which mimic that in Huh 7 cells, and 13 other p53 hot spot mutations (C141Y, V157F, G245S, R175H, R249S, R248Q, R273H, R280K, R282W, Y107A, H178A, 294delete and R342X) were obtained by site-directed mutagenesis from p53-WT-pcDNA3.1 plasmid with an addition of 3× Flag tag at the C-terminus. The wild-type p53 and mutant p53 plasmids were established by cloning the corresponding fragments into the pCDH-CMV-blast-sf3 (Systems Biosciences). Sequences of all primers used in the PCR-based cloning are provided in Table S1. To establish FKBP52 stable knockdown cells, pLKO.1 puro lentiviral vector (10878, Addgene) was used. Consistent data were obtained using distinct FKBP52-short hairpin (sh) RNAs, ruling out the possibility of off-target effects. The three sequences of FKBP52-shRNAs are listed in Table S1. The p53-shRNAs plasmids and MDM2-pcDNA3.1-HA were kind gifts from Professor Qingling Huang (Fujian Medical University). The cells were selected with 2 μg/ml puromycin (OGS541, Sigma-Aldrich) or 10 μg/ml blasticidin S (B9300, Solarbio) to establish stable expression cell lines. Plasmid DNA for transfection was purified with EndoFree Plasmid Maxi Kit (DP117, Tiangen) according to the manufacturer’s instruction.

### Cell transfection, lentivirus production, and transduction

Transfection in 293T, and Huh 7 cells was performed using polyethylenimine (PEI, 40816ES, YESEN) or Lipofectamine 2000 reagent (11668030, Thermo Fisher Scientific) according to the manufacturer’s protocol. For HepG2 cells and the cell lines used in the *in vivo* experiments, lentivirus were prepared and cells were stably transduced. Lentivirus production was prepared according to a commercial protocol (Addgene) with minor modifications. 293T packaging cells were cultured and three packaging plasmids (pGag/Pol, pRev, and pVSVG) together with pCDH-CMV-sf3 or pLKO.1-based transfer plasmids were diluted in DMEM containing 1 mg/ml PEI at a DNA: PEI ratio of 1: 3 to 1: 4. After 72 h, lentivirus were harvested and transduced according to the Addgene protocol. The cells were selected with puromycin (2 μg/ml) at least for 2 weeks.

### Immunohistochemistry

Tissue samples were fixed in 4% paraformaldehyde (PFA; AR1069, Boster, Wuhan, China) and embedded in paraffin. Primary antibodies against FKBP52 (ab124906, RRID:AB_10974784, Abcam), p53 (60283-2-Ig, RRID:AB_2881401, Proteintech, Wuhan, China), AFP (ab3980, RRID:AB_304203, Abcam), CD133 (18470-1-AP, RRID:AB_2172859, Proteintech), and p21 (10355-1-AP, RRID:AB_2077682, Proteintech) were used at a dilution of 1:200 according to the manufacturers’ protocols. Detection was performed using the Elite ABC Kit and 3,3′-diaminobenzidine (DAB) Substrate (PV-9000, ZSGB Biotech, Beijing, China), followed by hematoxylin counterstaining (105,175, Sigma-Aldrich).

Images were acquired using a BX53 microscope (Olympus) at 400 × magnification. The optical density of each tissue, represented by the integrated optical density (IOD), was calculated as: IOD = total area × mean density (MD). Quantitative analysis was performed using the Image-Pro Plus 6.0 image analysis system (Media Cybernetics), with three to five randomly selected fields analyzed per image.

### Cell growth assay

Cellular viability was determined using the cell counting kit 8 assay (CCK8, C0037, Beyotime) according to the manufacturer’s protocol. Briefly, Huh 7, HepG2 or Hep3B cells upon the indicated treatment were seeded in 96-well plates at a density of 2000 cells/well. At different time points, CCK-8 solution was added to each well and measured at 450 nm using a Cytation5 microplate reader (Biotek Instruments).

### Colony formation assay

For the clonogenic assay, human liver cancer cell lines were trypsinized to generate a single-cell suspension, which was seeded onto 35 mm dishes at a density of 2000 cells/well and cultured for 2 weeks. Colonies were washed twice with PBS, fixed with 4% paraformaldehyde (PFA) for 20 min at room temperature (RT), and stained with 0.1% crystal violet (C0121, Beyotime). Images were captured at 40 × magnification, and colony size and number were quantified using Image-Pro Plus 6.0 software.

### Soft agar assay

Approximately 2000 cells were suspended in a top layer of 0.8% agar (A9045, Sigma-Aldrich) and plated on a bottom layer of 1% agar in 6-well plates. After 4 to 6 weeks, colonies were photographed (40×) and counted. Scanned images of the plates were used to quantify colony number and size using ImageQuant TL (GE Healthcare).

### EdU incorporation assay

EdU (5-ethynyl-2′-deoxyuridine, a thymidine analogue, EdU-555, C0075, Beyotime) incorporation was used to quantify DNA synthesis during S phase. Briefly, cells were incubated with 10 μM EdU for 2 h at 37 °C, then fixed with 4% paraformaldehyde for 15 min and permeabilized with 0.3% Triton X-100 for 10 min. Click-iT chemistry was performed according to the manufacturer’s instructions. EdU-positive cells were visualized (ZEISS) and quantified as a percentage of total nuclei from at least five random fields per group.

### Confocal microscopy

Cells were seeded onto chamber slides (abs7027, Absin). After 24 h, cells were washed twice with PBS and fixed with 4% PFA for 20 min at RT. After washing, cells were permeabilized with 0.3% Triton X-100 in PBS for 10 min at RT, blocked with buffer (20% normal goat serum, 3% bovine serum albumin (BSA), 0.3% Triton X-100 in PBS) for 1 h at RT, then stained with a 1: 200 dilutions of primary antibody at 4 °C overnight, followed by simultaneous incubation with Alexa Fluor 488-conjugated goat anti-rabbit antibody (A-11094, RRID:AB_221544, Thermo Fisher Scientific) and Fluor-555 goat anti-mouse secondary antibody (A-21422, RRID:AB_2535844, Thermo Fisher Scientific) for 1 h at RT. Slides were mounted using VECTASHIED solution containing a 1: 2000 dilution of 4′,6-diamidino-2-phenylindole (DAPI, C1002, Beyotime). Images were acquired at 630× magnification using a high NA (1.49NA) water objective in the TCS SP8 confocal laser-scanning microscope (Leica), an A1R hybrid scan confocal microscope equipped with a high-resolution galvono scanner and an ultrahigh-speed resonant scanner (Leica), and a model LU4 four-laser acousto optic tunable unit filters with 405, 488, and 552 nm lasers equipped with LAS AF Lite imaging software.

### Western blotting and co-immunoprecipitation (Co-IP)

Cells lysates were obtained using radioimmunoprecipitation assay (RIPA) lysis buffer (50 mM Tris-HCl, pH 7.4, 150 mM NaCl, 0.5% Triton X-100, 1 mM phenylmethylsulfonyl fluoride) at 4 °C for 30 min. The cells were centrifuged at 12,000 rpm at 4 °C for 5 min and then boiled in SDS loading buffer for 5 min. Samples were separated by 8 to 15% SDS PAGE and transferred to polyvinylidene fluoride (PVDF) membranes which were blocked with buffer (5% skim milk or 3% BSA in Tris buffered saline containing 0.05% Tween 20) and incubated with primary antibodies to FKBP52; p53 (Rabbit, 10442-1-AP, RRID:AB_2206609, Proteintech); MDM2 (ab16895, RRID:AB_2143534, Abcam); HA-Mouse (M20003, RRID:AB_2864345, Abmart); HA-Rabbit (#3724, RRID:AB_1549585, Cell Signaling Technology [CST); Flag-Mouse (M20008, RRID:AB_2713960, Abmart); Flag-Rabbit (#8146, RRID:AB_10950495, CST); glyceraldehyde 3-phosphate dehydrogenase (GAPDH, #2118, RRID:AB_561053, CST) in blocking buffer.The membrane was probed with anti-mouse or anti-rabbit horseradish peroxidase-conjugated secondary antibody (#7076S, RRID:AB_330924, CST; #7074S, RRID:AB_2099233, CST), then developed with WesternBright ECL HRP substrate (K-12045-D50, Advansta).

For Co-IP, cells were lysed in RIPA buffer containing protease and phosphatase inhibitors (HY-K0010, MCE) for 45 to 60 min on ice, with brief vortexing every 10 min. The cells were centrifuged at 12,000 rpm for 10 to 20 min at 4 °C to remove cell debris. 5% cell lysates were kept as inputs, and other were immunoprecipitated with the corresponding antibodies for 6 to 12 h, followed by incubation with protein A/G beads (sc-2003, Santa Cruz Biotechnology) at 4 °C for 2 h. For the Flag-tagged protein, the cell lysates were directly incubated with M2 affinity magnetic beads (20565ES03, YESEN) for 4 to 6 h. The immunoprecipitants were washed two times each with RIPA and IP buffer before Western blot analysis.

### Cycloheximide (CHX) pulse-chase assay

Cells were seeded on 12-well plates at a density of 2 to 5 × 10^5^ cells/well and treated with 100 μg/ml CHX (239765, Sigma-Aldrich) dissolved in dimethylsulfoxide (DMSO). Cell lysates were collected at indicated time points.

### Statistical analyses

Statistical analyses were performed using Graphpad Prism 8.0 (RRID:SCR_002798). Data are shown as mean ± SD or mean ± SEM. Student’s t-tests (unpaired, 2-tailed) were used to compare two groups of independent samples. One- or two-way analysis of variance was used for multivariate comparisons where indicated. Survival analysis was performed using the Kaplan-Meier method, as assessed using a log-rank Mantel-Cox test. Significance is considered when ∗*p* < 0.05 and is indicated in the text as follows: ∗*p* < 0.05, ∗∗*p* < 0.01, ∗∗∗*p* < 0.001, ∗∗∗∗*p* < 0.0001. “ns” indicates no significant difference.

## Ethics approval

This clinical study and animal study were approved by the Institutional Review Board of Fujian Medical University, Fuzhou, China.

## Data availability

The data that support the findings of this study are included in this paper and its supplementary files.

## Supporting information

This article contains supporting information.

## Conflict of interest

The authors declare that they have no conflicts of interest with the contents of this article.
