# Supplementary material for: FKBP52 dictates the dual role of p53 in hepatocellular carcinoma: Mutation-specific prognostic stratification and therapeutic implications
Source: J Biol Chem. 2026 Aug 5;302(9):113390. doi: 10.1016/j.jbc.2026.113390 (PMC13553580; doi:10.1016/j.jbc.2026.113390)
Supplement: Supplementary file 1 [file mmc1.docx]

**Supplementary figures:**

Figure S1

**Supplementary Figure S1. FKBP52 expression in HCC grades. A,** Representative images of hematoxylin and eosin (H&E) staining and FKBP52 immunohistochemistry in #1 HCC tissue. Original magnifications were ×10 and ×40, and the scale bar denotes to 80 and 20 µm, respectively. Less invasive tumor nodules (benign areas) are outlined by a black line on the left. Black arrowheads point to giant cells. **B,** Quantification of FKBP52 staining intensity in Fig. 1E. Data are presented as mean ± SD, and t test was performed.

Figure S2

**Supplementary Figure S2. FKBP52 in-consistence in HCC cell lines of Huh 7 and HepG2. A,** Densitometric analysis of the Western blot shown in Figure 2A. **B** and **C,** Quantification of number (B) and area (C) upon FKBP52 overexpression in Fig. 2B. **D**, Densitometric analysis of the Western blot shown in Figure 2C. **E** and **F,** Quantification of colony number (E) and area (F) when knockdown of FKBP52 were used in Fig. 2D and 2E. **G**, Densitometric analysis of the Western blot shown in Figure 2F. **H,** Quantification of number and area of anchorage-**independent** colonies in HepG2 cells upon knockdown of FKBP52 in Fig. 2G. All experiments were repeated at least three times. Data are presented as mean ± SD. Two-tailed Student’s t tests or One-way ANOVA were used.

Figure S3

**Supplementary Figure S3. FKBP52 expression does not affect cell viability.** **A** and **B,** Quantification of cell viability based on CCK-8 measurement in Huh7 cells. **C** and **D,** Quantification in HepG2 cells. Data are presented as mean ± SD from triplicate measurements. Statistical significance was determined using One- or Two-way ANOVA.

Figure S4

**Supplementary Figure S4. shRNA-FKBP52 inhibits tumor burden in situ liver cancer model.** **A,** Gross images of tumor burden in the livers of mice as in Fig. 3F. **B,** Representative hematoxylin and eosin (H&E) staining and immunohistochemistry images of orthotopic tumors. Representative 10× images are shown, with the corresponding 40× higher-magnification views presented directly below. The regions shown at higher magnification are indicated by black boxes in the corresponding 10× images. The tissues were probed for the indicated antibodies. Scale bars denote 80 μm (10×) and 20 μm (40×). Representative images are shown.

Figure S5

**Supplementary Figure S5. Survival analyses of FKBP52 expression stratified by TP53 mutation status in HCC patients. A** and **B**, Progression-free interval (PFI) analysis of FKBP52 expression in TP53 wild-type (A) and TP53-mutant (B) hepatocellular carcinoma patients. **C** and **D,** Disease-free interval (DFI) analysis of FKBP52 expression in TP53 wild-type (C) and TP53-mutant (D) patients. The Kaplan–Meier analysis with log-rank testing were performed in (A), (B), (C) and (D).

Figure S6

**Supplementary Figure S6. FKBP52 regulates clonogenic and proliferative capacity in a p53-dependent manner. A** and **B**, Densitometric analysis of Western blot bands corresponding to Fig. 4E (A) and Fig. 4F (B)**. C and D,** Quantitative analysis of EdU incorporation assays in four HCC-related cell models. Panel C represents Huh7 and PLC/PRF/5 cells (Fig. 4G), while panel D represents HepG2 and HepaRG cells (Fig. 4H). **E,** Representative images of colonies from cells in Fig. 4I (original magnification). **F** and **G,** Quantification of colony number (F) and area (G) following p53 knockdown with or without FKBP52 overexpression in Huh 7, PLC/PRF/5 and HepG2 cells. **H**, Densitometric analysis of Western blot bands corresponding to Fig. 4J. Data are presented as mean ± SD from triplicate measurements. Statistical significance was determined using One-way ANOVA.

Figure S7

**Supplementary Figure S7. Densitometric quantification of immunoblot analyses for FKBP52-mediated regulation of p53 protein levels shown in Figure 5. A,** Quantitative analysis of FKBP52 protein levels corresponding to Fig. 5B. **B,** Quantitative analysis of p53 protein levels corresponding to Fig. 5C. **C,** Quantitative analysis of p53 protein levels corresponding to Fig. 5D. **D** Quantitative analysis corresponding to Fig. 5E. All experiments were repeated at least three times. Data are presented as mean ± SD. Two-tailed Student’s t tests or One-way ANOVA were used.

Figure S8

**Supplementary Figure S8. FKBP52–MDM2–p53 axis validation. A** and **B,** Quantitative analysis of wild-type p53 (L) and p53-Y220C mutant (M) in cells co-transfected with MDM2 and FKBP52. **C** and **D,** Quantitative analysis of MDM2 protein expression following FKBP52 overexpression or knockdown across two HCC-related cell models, including Huh7 and HepG2 cells. All experiments were repeated at least three times. Data are presented as mean ± SD. One, Two-tailed Student’s t tests or One-way ANOVA were used.
