# Supplementary material for: FKBP52 dictates the dual role of p53 in hepatocellular carcinoma: Mutation-specific prognostic stratification and therapeutic implications
Source: J Biol Chem. 2026 Aug 5;302(9):113390. doi: 10.1016/j.jbc.2026.113390 (PMC13553580; doi:10.1016/j.jbc.2026.113390)
Supplement: Supplementary file 2 [file mmc2.docx]

**S1 Table. Primers for cDNA cloning and siRNA sequences.**

|  | **Sequences (5′ to 3′)** |
| --- | --- |
| **Primers For cDNA cloning** | |
| FKBP52-pCDH-sf3-Flag F | GCTCTAGAGCCACCAtgacagccgaggag |
| FKBP52-pCDH-sf3-Flag R | TTGCGGCCGCCTACTTATCGTCGTCATCCTTGTAATCTGCTTCTGTCTCCAC |
| FKBP52-pcDNA3.1-HA F | GGGGTACCTTGCCACCATGACAGCCGAGGAG |
| FKBP52-pcDNA3.1-HA R | GCTCTAGATGCTTCTGTCTCCACCTGAGACT |
| FKBP52-pEGFP-N1-HA F | CGGCTAGCGCCACCATGACAGCCGAGGAGATG |
| FKBP52-pEGFP-N1-HA R | CCCTCGAGAGCGTAGTCTGGGACGTCGTATGGGTATGCTTCTGTCTCCACCTG |
| p53-Y220C-pcDNA3.1-HA F | CGGGATCCGCCACCATGGAGGAGCCGCAGTCA |
| p53-Y220C-pcDNA3.1-HA R | CGGAATTCGTCTGAGTCAGGCCCTTCTGTCTT |
| p53-WT-pcDNA3.1-HA F | CGGGATCCGCCACCATGGAGGAGCCGCAGTCA |
| p53-WT-pcDNA3.1-HA R | CGGAATTCGTCTGAGTCAGGCCCTTCTGTCTT |
| p53-WT-pCDH-sf3-Flag F | CGGAATTCGCCACCATGGAGGAGCCGCAGTCA |
| p53-WT-pCDH-sf3-Flag R | CGGGATCCGTCTGAGTCAGGCCCTTCTGTCTT |
| p53-Y220C-pCDH-sf3-Flag F | CGGAATTCGCCACCATGGAGGAGCCGCAGTCA |
| p53-Y220C-pCDH-sf3-Flag R | CGGGATCCGTCTGAGTCAGGCCCTTCTGTCTT |
| FK1-pcDNA3.1-HA F | TTGCGGCCGCGCCACCATGACAGCCGAGGAGATG |
| FK1-pcDNA3.1-HA R | GCTCTAGACTTAAACTCAAACAACTC |
| FK1+FK2-pcDNA3.1-HA F | TTGCGGCCGCGCCACCATGACAGCCGAGGAGATG |
| FK1+FK2-pcDNA3.1-HA R | GCTCTAGATTCAAAACTCTTGAGGTG |
| FK2+TPR-pcDNA3.1-HA F | TTGCGGCCGCGCCACCATGGGAGAAGATCTGACG |
| FK2+TPR-pcDNA3.1-HA R | GCTCTAGATGCTTCTGTCTCCACCTG |
| TPR-pcDNA3.1-HA F | TTGCGGCCGCGCCACCATGAAGGCCAAGGAGTCT |
| TPR-pcDNA3.1-HA R | GCTCTAGATGCTTCTGTCTCCACCTG |
| MDM2-pcDNA3.1-HA-F | AGGAGCAGGCAAATGTGCAATACCAACATGTCT |
| MDM2-pcDNA3.1-HA-R | TAGACAGGTCAACTAGGGGAAATAAGTTAGCAC |
| **Primers For p53 mutants construction** | |
| p53-R249S-F | gcatgaaccggagccccatcctcacca |
| p53-R249S-R | tggtgaggatggggctccggttcatgc |
| p53-V157F-F | ccggcacccgcttccgcgccatg |
| p53-V157F-R | catggcgcggaagcgggtgccgg |
| p53-R175H-F | gaggttgtgaggcactgcccccaccat |
| p53-R175H-R | atggtgggggcagtgcctcacaacctc |
| p53-R248Q-F | ggcggcatgaaccagaggcccatcctc |
| p53-R248Q-R | gaggatgggcctctggttcatgccgcc |
| p53-G245S-F | gttcctgcatgggcagcatgaaccggagg |
| p53-G245S-R | cctccggttcatgctgcccatgcaggaac |
| p53-R282W-F | cctgggagagactggcgcacagagg |
| p53-R282W-R | cctctgtgcgccagtctctcccagg |
| p53-C141Y-F | ccaactggccaagacctatcctgtgcagctgtgg |
| p53-C141Y-R | ccacagctgcacaggataggtcttggccagttgg |
| p53-R273H-F | gaacagctttgaggtgcatgtttgtgcctgtcctg |
| p53-R273H-R | caggacaggcacaaacatgcacctcaaagctgttc |
| p53-Y107A-F | cctaccagggcagcgccggtttccgtctgg |
| p53-Y107A-R | ccagacggaaaccggcgctgccctggtagg |
| p53-R280K-F | tgtgcctgtcctgggaaagaccggcg |
| p53-R280K-R | cgccggtctttcccaggacaggcaca |
| p53-H178A-F | gaggcgctgccccgcccatgagcgctgc |
| p53-H178A-R | gcagcgctcatgggcggggcagcgcctc |
| p53-294 delete-F | ccgcaagaaagggcctcaccacgagc |
| p53-294 delete-R | gctcgtggtgaggccctttcttgcgg |
| p53-R342X-F | cgagatgttctgagagctgaatgaggccttggaactcaa |
| p53-R342X-R | aagcgctcacgcccacggatctgaa |
| **siRNA sequences** | |
| FKBP52-pLKO.1-shRNA-1 | CGCAAGGACAAATTCTCCTTT |
| FKBP52-pLKO.1-shRNA-2 | GAAGAAGCTCTATGCCAATAT |
| FKBP52-pLKO.1-shRNA-3 | GCGGAATCATTCGCAGAATAC |
| p53-pLKO.1-shRNA-1 | CTCAGCATCTTATCCGAGT |
| p53-pLKO.1-shRNA-2 | GTCCCAAGCAATGGATGAT |
